# Composition analysis and antioxidant activity evaluation of a high purity oligomeric procyanidin prepared from sea buckthorn by a green method

**DOI:** 10.1016/j.crfs.2021.11.008

**Published:** 2021-11-25

**Authors:** Yulian Zhu, Michael Yuen, Wenxia Li, Hywel Yuen, Min Wang, Deandrae Smith, Qiang Peng

**Affiliations:** a College of Food Science and Engineering, Northwest A & F University, Yangling, 712100, China; b Beijing Engineering and Technology Research Center of Food Additives, Beijing Technology and Business University, Beijing, 100048, China; c Puredia Limited, No.12, Jing'er Road (North), Biological Technology Park, Chengbei District, Xining, Qinghai, China; d Department of Food Science and Technology, University of Nebraska, Lincoln Nebraska, USA, 68504

**Keywords:** Green preparation method, Oligomeric procyanidin, Synergistic antioxidant, Antioxidative stress activity, Sea buckthorn

## Abstract

Procyanidin is an important polyphenol for its health-promoting properties, however, the study of procyanidin in sea buckthorn was limited. In this paper, sea buckthorn procyanidin (SBP) was obtained through a green isolation and enrichment technique with an extraction rate and purity of 9.1% and 91.5%. The structure of SBP was analyzed using Ultraviolet–visible spectroscopy (UV–vis), Fourier-transform infrared spectroscopy (FT-IR), and liquid chromatography-mass spectrometry (LC-MS/MS). The results show that SBP is an oligomeric procyanidin, mainly composed of (−)-epicatechin gallate, procyanidin B, (+)-gallocatechin-(+)-catechin, and (+)-gallocatechin dimer. SBP showed superior scavenging capacity on free radicals. Furthermore, the cleaning rate of the ABTS radical was 4.8 times higher than vitamin C at the same concentration. Moreover, SBP combined with vitamin C presented potent synergistic antioxidants with combined index values below 0.3 with concentration rates from 5:5 to 2:8. SBP also provided significant protection against oxidative stress caused by hydrogen peroxide (H_2_O_2_) on RAW264.7 cells. These findings prove the potential of SBP as a natural antioxidant in food additives and support the in-depth development of sea buckthorn resources.

## Highlights

• A green method for the extraction of procyanidin was proposed.
• An oligomeric procyanidin in sea buckthorn was identified for the first time.
• SBP combined with VC exerted strong synergistic antioxidant.
• SBP provided protection of macrophages against oxidative damage.

## Graphical abstract

**Figure:**
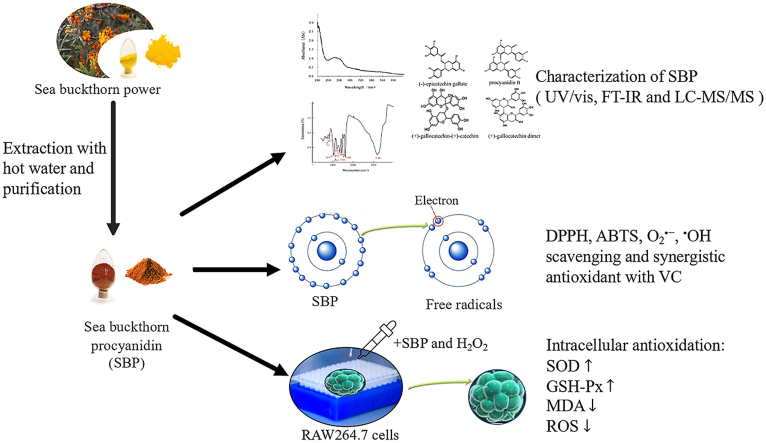

## 1 Introduction

Procyanidins are polyphenolic compounds found in fruits, tea, coffee, wine, chocolate, and, to a lesser extent, in some vegetables, cereals, and legume seeds. (Ashwin et al., 2021). Depending on the degree of procyanidins, they can be characterized as polymers or oligomers. Oligomeric procyanidins are mostly made of *(* + *)-catechin* and *(−)-epicatechin*, which are connected by C4–C6 or C4–C8 (Sui et al., 2016). Oligomeric procyanidins possess favorable biological properties due to their unique structure and low molecular weight. These properties include antibacterial, antioxidant stress and intestinal flora regulation abilities (Tang et al., 2017; Wu et al., 2021; Wang et al., 2019). Sea buckthorn is a shrub of the genus *Sour Spurge* in the family *Hochiaceae*. It is a traditional medicinal shrub widely cultivated in Europe and Asia (Ciesarova et al., 2020). Sea buckthorn berries are popular in pharmaceutical manufacturing due to a large number of active ingredients. It is abundant in vitamins, amino acids, and flavonoids (Mihalcea et al., 2018; Li et al., 2016; Xiao et al., 2021). Researchers have shown that the content and activity of active substances in sea buckthorn berries grown in different environments differ, with the berries of sea buckthorn grown at higher altitudes being of better quality than those grown in the plains (Guo et al., 2017).

In recent years, procyanidins found in the berries of sea buckthorn have attracted the interest of researchers due to their unique biological activity, beneficial potency, and low toxicity (Tkacz et al., 2021; Wang et al., 2020). According to available reports, methanol or ethanol were mainly used as solvents in the extraction of procyanidins (Pagano et al., 2021). These organic solvents had several disadvantages in industrial production, such as being highly toxic, flammable, harmful to the environment, and their low boiling point, which contradicts green production (Ruesgas-Ramón et al., 2017). Hence, developing a green and economical technology to extract sea buckthorn procyanidins is very important. Additionally, research on the active substances of sea buckthorn planted on plateaus was minimal, primarily on procyanidins. Thus, research on the extraction and activity of plateau sea buckthorn procyanidin was of particular significance for developing a new natural antioxidant.

The aim of this study is to extract procyanidin from plateau sea buckthorn by a green method, characterize its structural features, and evaluate its antioxidant activity in vitro through free radical scavenging and RAW264.7 cells model. The findings outline in the current research will provide theoretical support for the further utilization of sea buckthorn resources and develop sea buckthorn procyanidin as a natural antioxidant.

## 2 Material and methods

### 2.1 Materials and reagents

The sea buckthorn powder used for this study was obtained by Puredia Limited (Irvine, CA, USA), while the organic sea buckthorn was grown at an altitude of 2280–4622 m in Datong County, Qinghai Province, China. AB-8 macro-porous resin was purchased from Yunkai Resin Technology Co., Ltd Tianjin, China. RAW264.7 cells were obtained from the Shanghai Institution of Cell Biology (Shanghai, China) and the Dulbecco's Modified Eagle Medium (DMEM), and fetal bovine serum (FBS) were bought from American Type Culture Collection (ATCC, Manassas, VA). Other chemicals and solvents used in this study were of analytical grade.

### 2.2 Extraction and enrichment of sea buckthorn Procyanidin

Two times, hot water extraction (1:15 m/v, 55°C, 4 h) was applied to the sea buckthorn powder. After the treatment, the supernatant was filtered and concentrated at 55°C and −0.05 MPa. Subsequently, the AB-8 macro-porous adsorbent resin, using 30% ethanol as mobile phase at a flow rate of 150 L/h was used to enrich the concentrated solution (Puredia Limited, Irvine, CA, USA). Then the eluate was collected and a spray dryer (SP-1500, SUNYI TECH Shanghai, China) with the inlet temperature at 230 ± 5°C and outlet temperature at 94 ± 5°C was used to obtain the sea buckthorn procyanidin (SBP).

### 2.3 Purity determination

Standard solution of procyanidin at 95 μg/mL and 250 mg/mL SBP solution were prepared, and 1.0 mL of both standard solution and sample solution were dispensed to their respective vials. Then 6.0 mL of n-butanol hydrochloric acid mixture (95:5 v/v) and 0.25 mL of ammonium iron sulfate solution were added to each vial and mixed. The mixtures were heated at 95°C for 40 min. After the treatment, the vials were cooled in ice water for 15 min, divided into three parts, transferred to 10 mL volumetric flask, and diluted with the mixture of n-butanol and ethanol. The absorbances of solutions were measured with a spectrophotometer (model UV7, METTLER TOLEDO, Zurich Switzerland) at 551 nm and took methanol solution as the blank. Pure methanol was used as the blank. The purity of SBP was calculated using the following equation (Jerez et al., 2006):

$$Χ\%=\left(A_{1}/A_{0}\right)\times \left(V/W\right)\times 20\times 95\%$$

Where:

X% = the purity of sea buckthorn procyanidin
A_1_ = the absorption of sample solution
A_0_ = the absorption of standard solution
Cs = the concentration of the standard solution (mg/mL)
V = the volume of sample stock solution (mL)
W = the weight of sea buckthorn procyanidin (mg)

### 2.4 Characterization of sea buckthorn Procyanidin

#### 2.4.1 Fourier-transform infrared spectroscopy analysis

The FT-IR spectrum analysis of SBP was recorded on the Bruker-Vertex 70 Fourier transform infrared spectroscopy (FI-IR, Vetex70, Bruker Co., Ettlingen, Germany) using the KBr disk method at the scanning wavelength from 400 to 4000 cm^-1^.

#### 2.4.2 UV/vis analysis of SBP

SBP solution was prepared with a 100 mg/mL concentration and scanned at the 200–400 nm wavelength with a spectrophotometer (UV-2550 spectrophotometer, Mettler Toledo, Zurich Switzerland) (Fu et al., 2015).

#### 2.4.3 Analysis of liquid chromatography with tandem mass spectrometry

The sample of SBP (0.1 mg/mL) was filtered and analyzed by liquid chromatography with tandem mass spectrometry (LC-MS/MS) (Eroglu and Girgin, 2021). The analysis was conducted using an Agilent Zorbax eclipse C18 column (250 mm × 4.6 mm, 5 μM) equipped with an electrospray ion source and high resolution time-of-flight mass spectrometry (Triple TOF 5600, AB SCIEX, America). The mobile phases were 0.1% formic acid solution (A) and acetonitrile (B), the elution condition was carried as follows: 15% B, 0–5 min; 15%–20% B, 5–10 min; 25%–35% B, 20–30 min; 35%–50% B, 30–40 min; 80% B, 40–45 min; 15% B. The injection volume of SBP was 20 μL at the rate of 0.8 mL/min and the detection was operated at 30°C at a wavelength from 200 to 600 nm. The electrospray ionization (ESI) source was conducted under positive ion mode at a scanning rang from 100 to 1500 *m/z* and the specific parameters were as follows: the capillary voltage was 4.0 kV, the cone voltage was 20 V, the temperature of capillary voltage was 350°C and the collision energy was at 35%. The sheath gas and auxiliary gas had a flow rate of 30 arb and 5 arb.

### 2.5 Antioxidant activity determination in chemical assay

#### 2.5.1 DPPH radical scavenging ability

The DPPH radical scavenging ability method was based on a previous experiment conducted by Mekini et al. (2021). One mL of different concentrations of SBP solution (0.01–0.1 mg/mL) were prepared then mixed with 1 mL DPPH-ethanol solution (0.1 mmol/L). After being treated in the dark for 30 min at room temperature, each mixture's absorbance was measured at 715 nm by a spectrophotometer (UV7 spectrophotometer, METTLER TOLEDO, Zurich, Switzerland). Vitamin C (VC) solution (0.01 mg/mL) was used as the positive control.

#### 2.5.2 ABTS radical scavenging ability

Again, using a method by Mekini et al. (2021), with some modifications. Potassium persulfate (7.35 mmol/L) and ABTS radical solution (7 mmol/L) were mixed at the ratio of 1:2 (v/v) and stored at room temperature overnight as stock solution. The stock solution was diluted until its absorbance was 0.7 ± 0.02 at 734 nm to obtain ABTS radical working solution. Mixed 1 mL sample solution (0.5–10 μg/mL) with 3 mL diluted ABTS radical solution and incubated for 60 min at room temperature in the dark. After the treatment, the absorbances were measured at 734 nm with VC solution (10 μg/mL) as a positive control.

#### 2.5.3 Superoxide radical scavenging ability

The superoxide radical scavenging ability of SBP was determined using a method by Jerez et al. (2006). A 1 mL sample solution of SBP (0.01–0.1 mg/mL) was mixed with 4.5 mL Tris-HCl buffer (50 mmol/L pH 8.2) and 2.4 mL of deionized water, then added to 0.3 mL of 45 mmol/L pyrogallol and incubated for 1 h at room temperature. Subsequently, hydrochloride solution (HCl, 1.6 mol/L, 0.5 mL) was added to terminate the reaction. VC solution (0.01 mg/mL) was used as the positive control and the absorbances were measured at 325 nm.

#### 2.5.4 Hydroxyl radical scavenging ability

A methodology referenced in He et al. (2019) with slight modifications was used to determine hydroxyl radical scavenging ability. A 2 mL aliquot of sample solution (0.01–0.1 mg/mL) was mixed with 2 mL FeSO_4_ solution (9 mmol/L) and 2 mL salicylic acid-ethanol solution (9 mmol/L). After thorough mixing, hydrogen peroxide (H_2_O_2_, 2 mL, 8.8 mmol/L) was added to each mixture. The absorbances were then measured at 510 nm after 1 h of incubation at 37°C. VC solution (0.01 mg/mL) was used as a positive control.

The radical scavenging activity (RSA) was calculated using the following equation:

$$RSA\left(\%\right)=\left[1-\left(A_{sample}-A_{control}\right)/A_{blank}\right]\times 100$$

#### 2.5.5 Total reducing power determination

The method used by Odabasoglu et al. (2005) was used to determine the total reducing power of SBP. A 1 mL aliquot of sample solution (0.01–0.1 mg/mL) was mixed with 1 mL phosphate buffer solution (0.2 mol/L pH 6.6) and 2.5 mL potassium ferricyanide solution (1%, w/v), then reacted for 30 min at 50°C. After the treatment, 2.5 mL of trifluoroacetic acid solution (10%, w/v) was added to the mixture and shaken thoroughly.

The mixture was then centrifugated at 3000 rpm for 10 min, after which 2.5 mL of the supernatant was collected and mixed with 2.5 mL distilled water and 0.5 mL ferric chloride solution (0.1%, w/v) then vortexed for 1 min. The absorbance of each mixture was measured at 700 nm by the ultraviolet spectrophotometer and VC solution (0.01 mg/mL) was used as a positive control. The total reducing power was proportional to the absorbance.

#### 2.5.6 Evaluation of synergistic Antioxidant effects with vitamin C

The synergistic antioxidant activity of SBP and VC was investigated according to the reported method by Sharma et al. (2020). Different concentrations (0.01–0.1 mg/mL) of SBP and vitamin C mixture solution were prepared at the mass ratios of 8:2, 6:4, 5:5, 4:6 and 2:8, respectively. The synergistic antioxidant effects of SBP with VC were determined by assaying the DPPH scavenging ability and total reducing power. Based on Talady and Chou's mesophilic principle, combination index (CI) was applied to evaluate the synergistic antioxidant effects via the following equation:

CI = D_1_ / D_X1_ + D_2_ / D_X2_

Where:

D_1_ and D_2_ (mg/mL) were the fractional inhibitory concentration fifty percent indexes compound 1 and compound 2 in the mixtures.
D_X1_ and D_X2_ (mg/mL) were the fractional inhibitory concentration fifty percent indexes of compounds 1 and 2 when they act separately.
CI < 1 indicates synergistic antioxidant effects
CI = 1 indicates superimposed antioxidant effects
CI > 1 indicates antagonistic antioxidant effects
0.7 < CI < 1 indicates slight coordination antioxidant effects
0.3 < CI < 0.7 indicates general synergistic antioxidant effects
CI < 0.3 indicates strong synergistic antioxidant effects

### 2.6 Protection on RAW264.7 cells against oxidative damage

#### 2.6.1 Cytotoxicity of sea buckthorn Procyanidin on RAW 264.7 cells

This experiment referenced to the study by Zhu et al. (2021). The RAW264.7 cells (5.0 × 10^4^ cell/mL) were obtained and incubated under the condition of 5% CO_2_ atmosphere condition for 24 h at 37°C in the 96-well plates. Subsequently, different concentrations of SBP (25, 50, 100, 200, 400, 800, 1000 μg/mL) were added to incubate the cells, culture medium and lipopolysaccharide (LPS) at the 1 μg/mL concentration and were used as the negative and positive control groups, respectively. After 24 h incubation, 10 μL CCK-8 solution was added to each group and continuously incubated for another 1 h. An ELISA reader was used to measure the cell viability at 450 nm (victorX3, PerkinElmer Co., Waltham, Massachusetts, US).

#### 2.6.2 Establishment of the H_2_O_2_ oxidative damage model and the observation of cell morphology

RAW264.7 cells (5.0 × 10^4^ cells/mL) were incubated in 96-well plates at the condition of 5% CO_2_ at 37°C for 24 h. After which, the medium was removed, and different concentrations of H_2_O_2_ (0, 200, 300, 400, 500, 600, 700, 800, 900 μmol/L) diluted with RPMI-1640 culture medium were added to each group (Navarro-Hoyos et al., 2018). The cell viability was detected using the Cell Counting Kit-8 (CCK-8, EnoGene Co., Shanghai, China) after 4 h of incubation. At the same time, the morphology of the cells after treatments was observed using an inverted microscope (IX71S1F-3 OLYMPUS CORPORATION, TOKYO, JAPAN).

#### 2.6.3 Protection of sea buckthorn Procyanidin on oxidative stress

RAW264.7 cells (2.0 × 10^5^ cells/mL) were incubated in 96-well plates at 37°C for 24 h, then removed from the medium and exposed to different concentrations (25, 50, 100 μg/mL) of SBP solution for 4 h. Subsequently, 800 μmol/L of H_2_O_2_ was added to sample groups and incubated for another 4 h. The positive control group was treated with 25 μg/mL of VC solution instead of SBP. The CCK-8 assay was used to determine the cell viability (Sierra-Cruz et al., 2021).

#### 2.6.4 Determination of superoxide dismutase, Glutathione peroxidase activities and malon-dialdehyde level

RAW264.7 cells treated with different concentrations of SBP or VC were collected for the analysis of antioxidant enzymes. Superoxide Dismutase (SOD) activity was measured using a commercially bought kit (Beyotime, Biotechnology, Shanghai, China) and determined as the standard assay. and determined as the standard assay. Glutathione peroxidase (GSh-Px) activity was measured by the relevant commercial kits and measured using a method referenced by Zhang et al. (2021). The level of malon-dialdehyde (MDA) was detected with an assay kit (S0131S Betotime Biotechnology) following the manufacturer's instructions.

#### 2.6.5 Determination of intracellular reactive oxygen species

The RAW264.7 cells (1.0 × 10^5^ cells/mL) were seeded in 96-well plates and incubated with different concentrations of SBP (25, 50 and100 μg/mL) and of VC (25 μg/mL) for 4 h, followed by the addition of 800 μmol/L H_2_O_2_ and treated for another 4 h. The intracellular reactive oxygen species (ROS) was detected using a ROS detection kit (CA1410 Sobolite Betotime Biotechnology). The intracellular scavenging ability was measured by the F-7000 fluorescence spectrophotometer (Pepro Tech, Cranbury, New Jersey, USA).

### 2.7 Statistical analysis

A one-way fixed-effects analysis of variance (ANOVA) test was performed using statistical software (SPSS version 18.0, SPSS Inc., Chicago, IL, USA). All trials were done in triplicate, and the statistical means and standard deviations were calculated and shown.

## 3 Results and discussions

### 3.1 Preparation of sea buckthorn Procyanidin

**Fig. 1:**
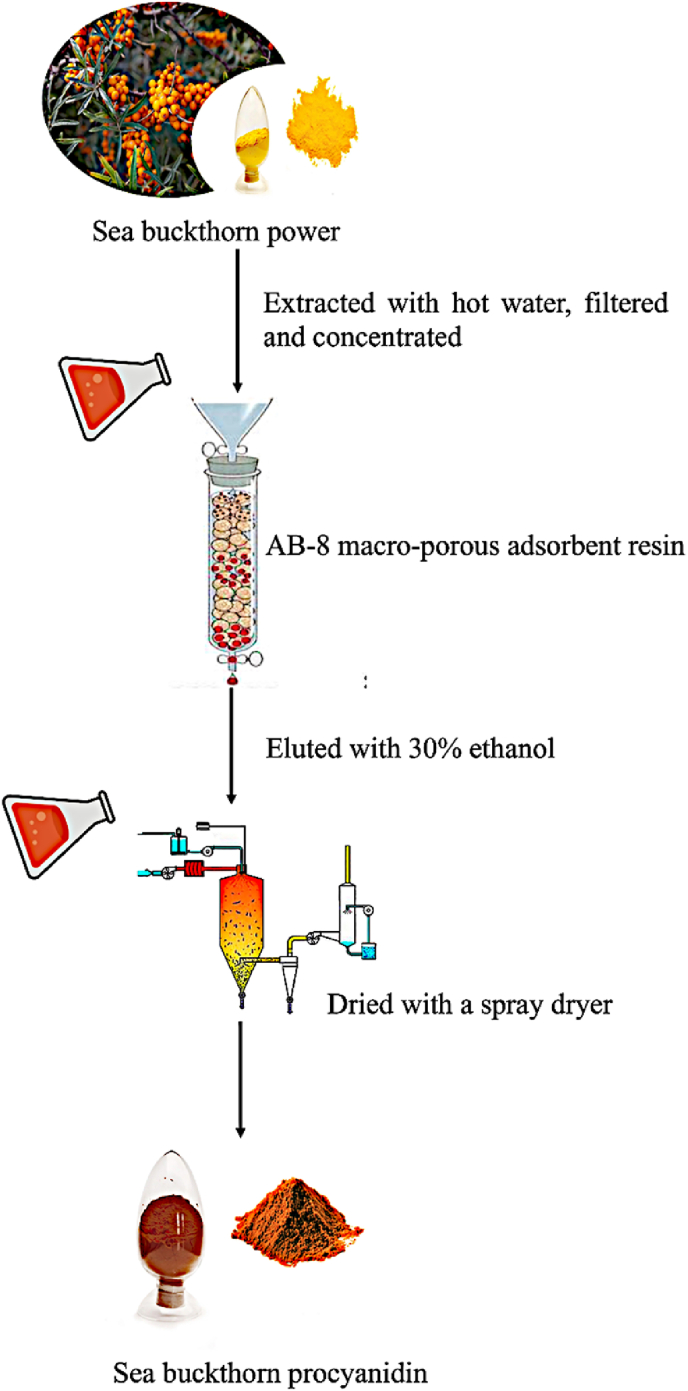
The flow chart of the preparation of Sea Buckthorn Procyanidin.

The flow chart of the extraction and enrichment of SBP is shown in Fig. 1. The crude extract of sea buckthorn powder was extracted with hot water and vacuum concentration. The hot water extract was under the trade name of Cyanthox. Then mixture was then enriched using macro-porous resin, using ethanol as the eluent to obtain SBP. SBP was obtained by spray drying the pure extraction solution, and the yield of SBP was 9.1%. The purity test results showed that the purity of SBP was 91.5%, indicating very high purity and the excellent enrichment. This can be compared to the procyanidins extracted from sea buckthorn with 80% ethanol at the content of 31.9% in the ethanol extract (Zhang et al., 2018). It is believed that the high quality of SBP extracted in this present study was due to the sea buckthorn being planted on the plateau, the exceptional temperature, altitude environment, light intensity, and the organic culture method used.

### 3.2 Characterization of sea buckthorn Procyanidin

#### 3.2.1 Analysis of ultraviolet–visible spectroscopy and Fourier-transform infrared spectroscopy

**Fig. 2:**
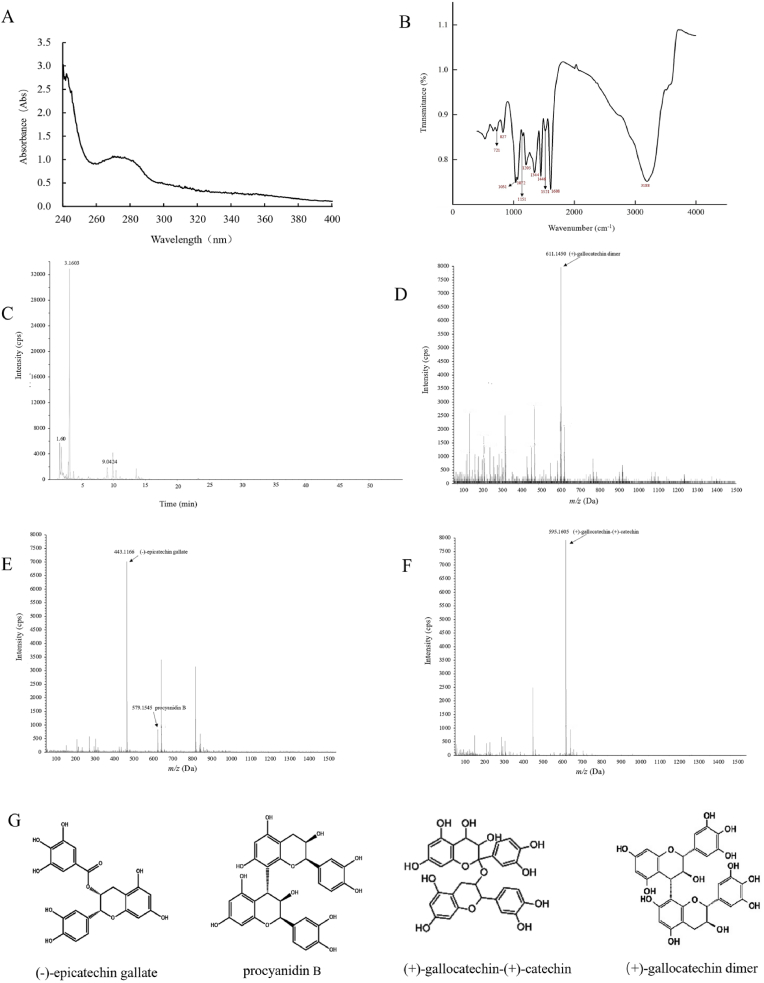
(A) The UV/vis spectrum; (B) The FT-IR spectrum of sea buckthorn procyanidin; (C) The total ion chromatogram of sea buckthorn procyanidin; (D) MS spectra of compounds eluted at 1.617 min; (E) MS spectra of compounds eluted at 3.1603 min; (F) MS spectra of compounds eluted at 9.0424 min; (G) The structure of compounds detected through MS.

The SBP solution showed pronounced characteristic absorption peaks at 280 nm, which was the typical characteristic of procyanidins (Fig. 2A). It is suspected that the 280 nm peak was caused by the conjugated structure of benzene rings of SBP. This peak was also similar to the full-wavelength scanning spectrum of catechins, indicating that the main constituent unit of SBP may be catechins and that SBP was a typical polyphenol (Yusoff et al., 2020).

From the infrared spectrum of SBP (Fig. 2B), it could be concluded that there was a strong band at 3188 cm^-1^, which might be due to the broad hydroxyl stretching vibration of the phenolic structure in SBP (Xu et al., 2012). Peaks at 1608 cm^-1^ corresponded to the C=O stretch vibration, suggesting the existence of galloyl groups. Vibration absorption peaks at 1521 cm^-1^, 1446 cm^-1^, and 1344 cm^-1^ were generally assigned to the structure of aromatic compounds in SBP, and the absorption at 1205 cm^-1^ was due to the vibration of aromatic rings (Munoz-Labrador et al., 2019). The absorption band at 1115 cm^-1^ was due to the C–H stretching vibration in the C ring of the procyanidins unit (Zou et al., 2012). Peaks 1072 cm^-1^ and 1031 cm^-1^ were due to the C–C stretching vibration in the procyanidins molecule, and 827 cm^-1^ and 721 cm^-1^ were due to the unsaturated C–H out-of-plane deformation vibration of the aromatic ring (Yusoff et al., 2020). It could be seen that the structure of SBP had distinct functional groups of procyanidins, which were consistent with research by Munoz-Labrador et al. (2019) on the procyanidins-rich fraction extraction of grape seed.

#### 3.2.2 Analysis of liquid chromatography with tandem mass spectrometry

LC-MS/MS is a rapid technology for identifying and analyzing natural compounds by measuring ions obtained during HPLC separation, and through the mass spectrometry results and the analysis of MS/MS fragments, the main compound *m/z* of each peak and its fragment ion *m/z* could be obtained, and the structural unit and possible compounds could be effectively analyzed (Rue et al., 2018). In this study, the further evaluation of SBP was conducted using an LC system MS equipped with an ESI source with a positive ion mode. The chromatogram and mass spectrum are shown in Fig. 2. The overview of the composition of SBP was given in Table 1, and the analytical results of the components were summarized along with their *m/z* experimental, MS/MS fragments and formula. The specific structure of all the existed components were shown in Fig. 2G. From the chromatographic in Fig. 2C, it could be seen that the peaks of each composition of SBP were separated within 10 min, among which the compounds at 1.617 min, 3.1603 min, and 9.0424 min were relatively high. The specific compounds of each peak needed to be further identified by mass spectrometry. In general, in order to distinguish procyanidins structure, a nomenclature based on the position of rings in procyanidins was developed, the T-unit was on the top of the structure and with the linkage at C4, M-unit was in the middle with the linkage at C4 or C8, the unit with three C–C linkage was called J-unit and the C–C linkage at C8 or C6 was B-unit (Friedrich et al., 2000). Quinone methide (QM), retro Diels-Alder (RDA), Heterocyclic Ring Fission (HFR), and break of bands between flavonoids were the main pathways of procyanidins fragmentation (Rue et al., 2018). QM fragmentation assisted in the cleavage of procyanidin polymer and contained two molecules of catechins or epicatechins and resulted in several different ions. The HRF process was a pathway that occurred on the units of procyanidin B dimers, and RDA was the most common fragmentation pathway of procyanidins resulting in the break of B-type procyanidins (Hellstrm et al., 2007).

**Table 1:** Liquid chromatography and mass spectral characteristics of SBP.

Compound	[M+H]^+^ (*m/z*)	MS/MS	Formula	Identification
C1	443.11	333, 291, 273, 247, 171	C_22_H_18_O_10_	(−)-epicatechin gallate
C2	579.15	453, 427, 409, 292, 289, 247	C_30_H_26_O_12_	procyanidin B
C3	595.16	469, 443, 425, 409, 307	C_30_H_26_O_13_	(+)-gallocatechin-(+)-catechin
C4	611.14	593, 485, 443, 425, 307, 303	C_30_H_26_O_14_	(+)-gallocatechin dimer

The mass spectrometry of peak 1 eluted at 1.617 min is shown in Fig. 2D. The major molecular ion [M+H]^+^ was 611 *m/z*, and its MS/MS fragments were 593, 485, 443, 425, 307, and 303. The *m/z* of the fragment at 593 was [M-H2O–H], producing by missing an H_2_O from the molecule at the *m/z* of 611. The fragment at 485 *m/z* was regarded as [M-C_6_H_6_O_3_-H]^+^, which was produced by removing a molecule of phloroglucinol through the HFR fragmentation pathway. The RDA fragmentation pathways of 611 *m/z* resulted in fragments of [M-C_8_H_8_O_4_-H]^+^ at 443 *m/z* and [M-C_8_H_8_O_4_-H]^+^ further eliminated another H_2_O molecule to arise [M-C_8_H_8_O_4_-H_2_O–H]^+^ at the *m/z* of 425. Besides, the fragment at 307 *m/z* and 303 *m/z* were regarded as [M_B_-H]^+^ and [M_T_-H]^+^, which were caused by the cleavage of flavonoid linkages through QM fragmentation and the RDA fission in the B unit (Friedrich et al., 2000). Thus, the molecule at 611 *m/z* could be identified as a (+)-gallocatechin dimer. The dimer at the *m/z* of 595 was consisted of the fragments at 469, 443, 427, 425, 409, and 307 (Table 2). It could be concluded that the fragment at 469 *m/z* was [M-C_6_H_6_O_3_-H]^+^, probably resulting from the loss of the loss of B ring through the HRF cleavage of 595 *m/z*. The 443 and 425 m/z was regarded as [M-C_8_H_8_O_3_-H]^+^ and [M-C_8_H_8_O_4_-H]^+^, which arise from the fission of B and T units through RDA fragmentation. Dehydration of phenolic hydroxyl groups could be inferred from the processes of 427 to 409 *m/z* ([M-C_8_H_8_O_4_-H_2_O–H]^+^) (Liu et al., 2013). Accordingly, the QM pathway broke the flavonoid linkages and resulted in the fragment at 307 *m/z* ([M_T_-3H]^+^). Thus, 595 *m/z* was classified as a dimeric fragment consisted (+)-gallocatechin and (+)-catechin (Kiselova-Kaneva et al., 2022). The signal at 579 *m/z* could be regarded as a B-type procyanidin dimer. DRA fragmentation was the most common pathway of B-type procyanidin dimer, and RDA pathway of 579 *m/z* produced the fragment of [M-C_8_H_8_O_4_-H]^+^ at the *m/z* of 427. The fragment ion of [M-C_6_H_6_O_3_-H]^+^ at 453 *m/z* might produce by the HRF fragmentation of B-type procyanidin dimer. The fragment at 427 *m/z* further dehydrated to produce 409 *m/z* ([M-C_8_H_8_O_4_-H_2_O–H]^+^). QM fragmentation and the decomposition of flavonoid linkages in procyanidin B resulted in the loss of T-unit and the fragment at 291 *m/z* was regarded as [M_B_-H]^+^, the fragment [M_B_-CO_2_-H]^+^ at 247 *m/z* was produced by removing a molecule of CO_2_ of 291 *m/z* (Silva et al., 2021). (−)-Epicatechin gallate was detected as the major fragment eluted at the speak of 3.1603 min and the mass spectrometry can be seen in Fig. 2E at the *m/z* of 443.11. The MS/MS results of 443.11 *m/z* showed the fragments at 333, 291, 273, 247 and 171 *m/z*. The fragment at 333 *m/z* might be [M-C_6_H_6_O_2_-H]^+^, which was caused by removing the B ring from (−)-epicatechin gallate, 291 ([M-C_7_H_6_O_5_-H]^+^) was produced by loss of galloyl group，and 273 *m/z* ([M-C_7_H_6_O_5_-H_2_O–H]^+^) might be caused by losing a molecule of H_2_O from 291 *m/z* (Sasot et al., 2017). The fragment at 247 *m/z* was [M-C_7_H_6_O_5_-H_2_O–H]^+^ and was produced form the further dehydration of [M-C_7_H_6_O_5_-H]^+^. It was evident that the fragment at 171 *m/z* was the presence of C_7_H_6_O_5_, which was the present of gallic acid, the typical group of (−)-epicatechin gallate (Stalmach et al., 2011).

**Table 2:** Combination Index values of the DPPH radical scavenging.

Proportion (SBP: VC)	8:2	6:4	5:5	4:6	2:8
CI values	0.396 ± 0.021	0.337 ± 0.035	0.296 ± 0.051	0.251 ± 0.027	0.142 ± 0.034
Synergy level	general	general	strong	strong	strong

From the results of the mass spectra, the corresponding substances *m/z* were all less than 800, and most of the components were dimers, which suggested that SBP was a typical phenolic polymer with a low degree of polymerization (Bai et al., 2019). In addition, the monomeric phenolics that made up SBP were also consistent with the FT-IR results. According to relevant reports, the lower degree of procyanidins might also endow SBP with unique biological activity (Navarro-Hoyos et al., 2018).

### 3.3 Antioxidant activity in chemical methods

#### 3.3.1 DPPH radical scavenging ability

**Fig. 3:**
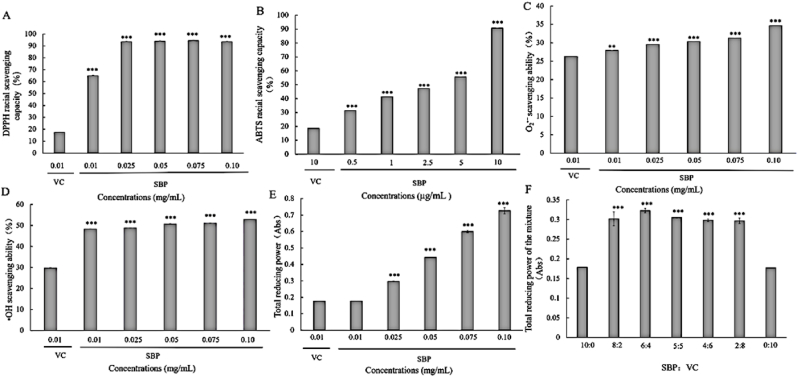
Antioxidant of SBP in chemical methods: (A) DPPH radical scavenging capacity; (B) ABTS racial scavenging capacity; (C) O_2_^•−^ scavenging ability; (D) ^•^OH scavenging ability; (E) Total reducing power; (F) Total reducing power of the mixture. (*) p < 0.05, (**) p < 0.01, (***) p < 0.001.

DPPH radical scavenging ability was the most convenient method to measure the antioxidant capacity in vitro, and it is a reproducible method frequently reported to confirm the antioxidant capacity of biomolecules, phenols, and food. The DPPH radical was stable due to the conjugation and site-blocking of the benzene ring and its electron-absorbing effect of nitro (Yang et al., 2021). DPPH exhibited a dark purple color in ethanol and produced a maximum absorption at 516 nm. The absorbance would decrease by adding radical scavengers such as antioxidants (Spranger et al., 2008). The DPPH radical scavenging capacity by SBP and VC is shown in Fig. 3A. The DPPH radical scavenging capacity ranged from 47.92% to 94.56% when the concentrations of SBP increased from 0.01 to 0.10 mg/mL. Compared to the positive control at the scavenging rate of 17.5%, the results indicated that SBP showed significant clearance rates of DPPH radical, and the capacity was also enhanced with the increasing dose. The successful DPPH radical scavenging capacity may be related to the C=C bond in the benzene ring of SBP. The phenolic hydroxyl group tends to confer higher antioxidant properties to procyanidins due to the extension of the conjugated structure on the benzene ring (Yang et al., 2021).

#### 3.3.2 ABTS radical scavenging ability

ABTS was oxidized to green ABTS radical by the oxidant potassium persulfate and had a maximum absorption peak at 734 nm in UV–visible light (Spranger et al., 2008). Therefore, it was possible to establish a colorimetric method for supplementing light and oxidation resistance and changing the absorbance value at 734 nm. In this study, the ABTS radical scavenging ability was evaluated by the low concentration of SBP (0.5–10 μg/mL) and compared with the VC solution at 10 μg/mL. Results exhibited a significant scavenging ability of SBP in scavenging ABTS radicals in different concentrations compared to VC solution (18.61%) (Fig. 3B). The scavenging ratios were from 31.4% to 90.71% with the increase of samples concentrations. The scavenging capacity of SBP was even 4.8 times higher than that of VC at the concentration of 10 μg/mL. Antioxidant activity measurement in chemicals always exhibited a lower capacity evaluated by DPPH assay relative to the ABTS method. The reason might be that the evaluation of ABTS·^+^ was more suitable for hydrophilic and lipophilic systems (Kim et al., 2021). Thus, the antioxidant activity of procyanidins could be compared quickly and effectively through ABTS assay.

#### 3.3.3 Superoxide radical scavenging ability

In weakly alkaline conditions, o-triphenol could undergo an autoxidation reaction to produce superoxide anions and colored intermediate products, with a characteristic absorption peak at 320 nm. The number of intermediate products had a linear relationship with time. When the superoxide anion scavenger was added, it rapidly reacted with the superoxide anion, thus preventing the accumulation of intermediates and decreasing the absorbance of the solution at 320 nm (McCord and Fridovich, 1969). Therefore, the scavenging effect of the scavenger on superoxide anion could be evaluated by measuring the absorbance at 320 nm. As shown in Fig. 3C, the scavenging rates of SBP on superoxide free radicals ranged from 27.95% to 34.65% with the increase of SBP concentrations. The overall clearance effects in this indicator were slightly lower than the free radical scavenging ability of DPPH and ABTS. It can be surmised that the superoxide radical was mainly scavenged by intracellular superoxide dismutase, while the scavenging capacity was limited in vitro chemical methods (Azmi et al., 2019).

#### 3.3.4 Hydroxyl radical scavenging ability

With the addition of salicylic acid, the hydroxyl radicals generated by the Fenton reaction in the system reacted with salicylic acid to produce 2,3-dihydroxybenzoic acid with unique adsorption at 510 nm (Yang et al., 2010). When the hydroxyl radical scavenger was added, the number of hydroxyl radicals generated would reduce and thus decrease the production of colored compounds generated in the solution. The absorbances of the reaction solution containing SBP were measured using the fixed reaction time method and compared with the blank solution to determine the scavenging effect of SBP on hydroxyl radicals. As presented in Fig. 3D, the hydroxyl radical scavenging capacity of SBP increased from 48.3% to 52.91% with the change of concentrations. At the same concentration of 0.01 mg/mL, SBP (48.3%) exhibited a significantly higher scavenging effect than VC (29.82%), which indicated the high potential of SBP for applications in the pharmaceutical and food industries.

#### 3.3.5 Total reducing power determination

Antioxidants could reduce Fe^2+^ to Fe^3+^ effectively, which could chelate with potassium ferricyanide to form a blue solution with a maximum absorption peak at 700 nm (Liu et al., 2021). Therefore, the total reduction capacity could be determined by establishing the SBP systems and comparing the absorption changes at 700 nm. The reducing power of SBP and the positive control are shown in Fig. 3E. SBP samples represents a dose-dependent manner, and the absorbance values of different concentrations (0.01–0.10 mg/mL) were 0.177–0.727 Abs. Moreover, the absorbances of all SBP samples were higher than VC solution, indicating that SBP had a significant antioxidant effect. Similarly, procyanidins obtained from lychee pericarp also exhibited great reducing power. Combined with the radicals scavenging capacity, it could be concluded that SBP was a biologically active molecule with a high-efficiency antioxidant effect (Luo et al., 2020).

#### 3.3.6 Evaluation of synergistic Antioxidant effects with vitamin C

The antioxidant effect of the combination of multiple antioxidants was significantly higher than that of a single antioxidant at the same dose. Synergistic effects between antioxidants were influenced by the ratio, concentration, and type of compounds (Quiroga et al., 2019). The study of synergistic effect between SBP and VC was essential for evaluating the potential of SBP to be an effective compound natural antioxidant. The synergistic antioxidant results of the combination of SBP and VC at different concentration ratios are exhibited in Table 2 and Fig. 3F. In DPPH radical scavenging ability and total reducing capacity assays, the combined index (CI) values varied with the proportions of the compounds. It could be concluded that the combination showed a synergistic effect because the CI values were consistently below 1. At the ratio at 8:2 and 6:4, the CI values ranged from 0.3 to 0.7, which indicated a general synergism. At the ratio of 5:5 to 2:8, the CI values were all below 0.3 and represented a strong synergism. The consistent effect was also implied by the conclusions of the total reducing power. In Fig. 3F, the total absorbances varied with the ratios of SBP:VC. The compounds showed higher absorbance values when SBP content was higher than VC, while the absorbances decreased as the VC increased. Such a trend suggested a dominant role for SBP in the compounds’ antioxidant capacity. Absorbances of all compound groups were higher than that of SBP and VC working alone, which confirmed the synergistic antioxidant properties of the compounds. In the compound system, VC was a potent antioxidant, and it provided SBP with hydrogen atoms to regenerate its antioxidant activity, resulting in the great synergistic antioxidant when SBP was combined with VC (Hu et al., 2021). The results were consistent with the results of studies on synergistic antioxidants of medicinal vegetable oils reported in the literature, demonstrating that SBP combined with VC was an effective compound antioxidant (Sharma et al., 2020).

### 3.4 Resistance to oxidative damage in RAW264.7 cells

#### 3.4.1 The cytotoxicity of SBP to RAW 264.7 cells

**Fig. 4:**
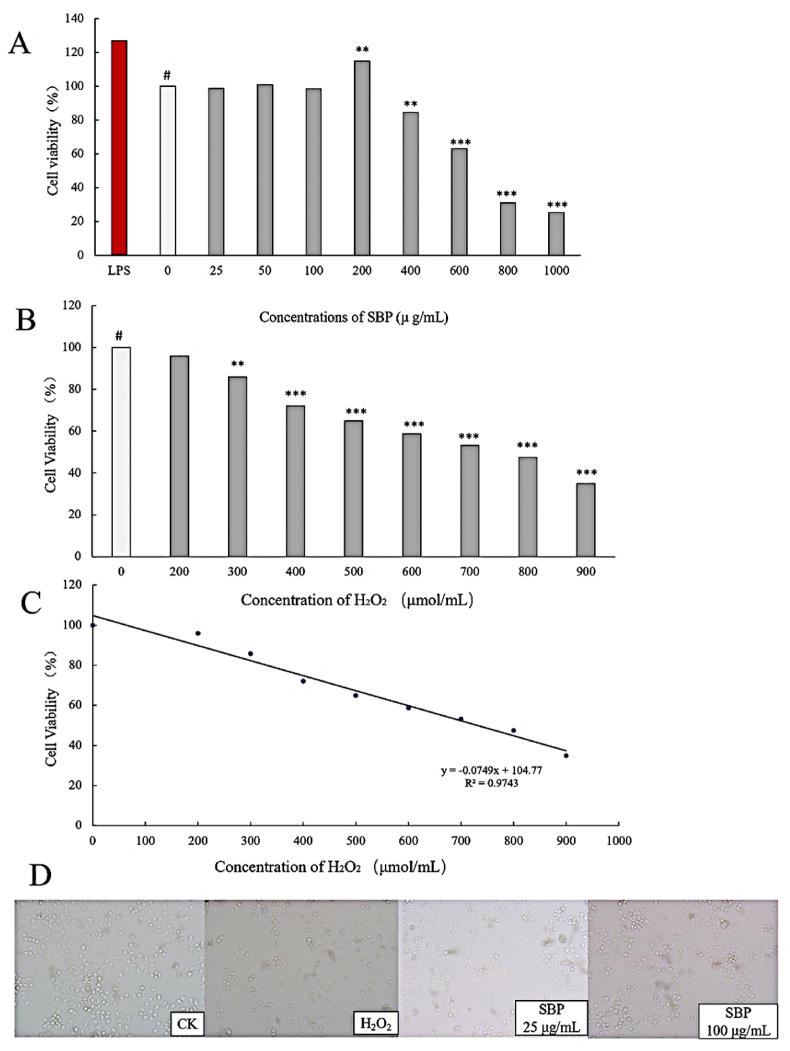
(A) Cell viability of RAW264.7 cells in the cytotoxicity test of SBP; (B) Cell viability of RAW264.7 cells with different concentrations of H_2_O_2_; (C) The linear regression equation of RAW264.7 cells viability; (D). Cell morphology of each treatment.

RAW264.7 cells were treated with SBP at the doses of 25, 50, 100, 200, 400, 800 and 1000 μg/mL to determine the influence of the samples’ cytotoxicity. This was determined by the cells’ viability. It could be concluded from Fig. 4A that the toxic effects of SBP at a concentration of 20–100 μg/mL on RAW264.7 cells were not significant. However, the toxic effect was evident when the concentrations exceeded 200 μg/mL. Hence, the maximum concentration of SBP at 100 μg/mL was selected to reduce the interference of SBP in the following study.

#### 3.4.2 Establishment of oxidative damage model by H_2_O_2_ and observation of cell morphology

RAW264.7 cells were incubated with different concentrations of H_2_O_2_, and suitable doses were selected to establish an oxidative stress model. The cell viability results were calculated using the MTT method and shown in Fig. 4B. As the H_2_O_2_ concentrations increased to 300 μmol/L, cell viability decreased significantly, indicating the oxidative stress state of cells. Additionally, the linear regression equation showed the IC_50_ was 785.2 μmol/L in Fig. 4C. Thus, the concentration of H_2_O_2_ at 800 μmol/L was chosen as the oxidation damage condition in the subsequent experiments.

As shown in Fig. 4D, the density of damaged RAW264.7 cells in the microscope field decreased after treatment of SBP. Compared with the cells incubated with H_2_O_2_, sample groups showed a higher density and better morphology. It could be explained that oxidative stress induced by H_2_O_2_ led to cellular damage, while SBP presented resistance to the toxicity. However, these results could not accurately indicate the antioxidant activity of SBP. Therefore, the changes of various intracellular indicators and cell viability requires further investigation.

#### 3.4.3 Protective effect against oxidative damage by H_2_O_2_

**Fig. 5:**
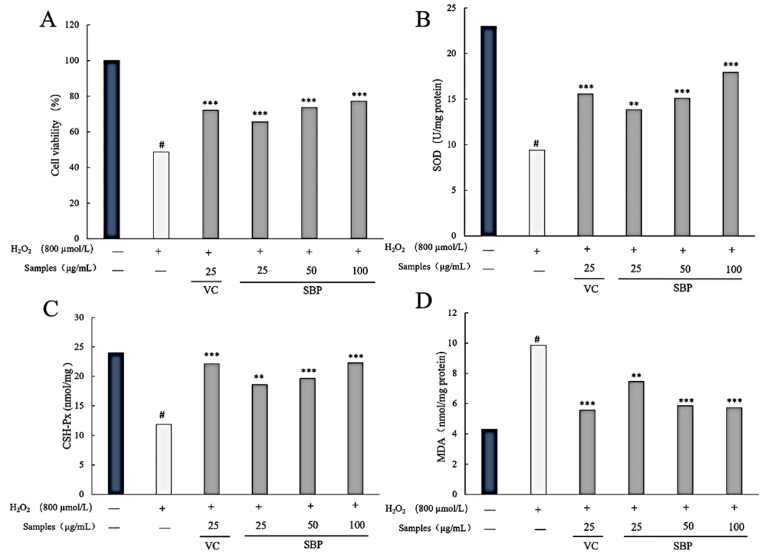
(A) Cell viability of RAW264.7 cells with the protection of SBP; (B) SOD content of cells with different treatments; (C) GSH-Px content of cells with different treatments; (D) MDA content of cells with different treatments.

From Fig. 5A, groups with the addition of VC and SBP were highly significant in inhibiting cell death caused by H_2_O_2_, and the cell viability increased by 34.86%, 51.28% and 58.59% with the change of SBP concentrations (25, 50, 100 μg/mL) compared to the H_2_O_2_ group, respectively. At the concentration of 25 μg/mL, the cell viability of the SBP group was lower than the VC group, which was inconsistent with those of the chemical experiments. This phenomenon could be explained by the influence of many factors on cellular anti-oxidation. In addition to the ability of the samples themselves to scavenge free radicals, it was also related to various factors such as improving cellular antioxidant enzyme activity, reducing intracellular reactive oxygen species levels (Mizuno et al., 2019), protecting mitochondrial function (Su et al., 2018), and delaying apoptotic signaling pathways (Yan et al., 2021).

#### 3.4.4 Determination of SOD activity, GSH-Px level and MDA content in RAW264.7 cells

There were two defense systems, enzymatic and nonenzymatic, used to eliminate or reduce the oxidative damage caused by free radicals. The enzyme defense system includes superoxide dismutase (SOD) and glutathione peroxidase (GSH-P_X_), which have been selected when assessing the antioxidant activity of natural products in vitro (Olsvik et al., 2005). SOD was an essential enzyme in antioxidant systems which could catalyze the conversion of superoxide anion to H_2_O_2_. GSH-Px could scavenge H_2_O_2_ directly, thus blocking lipid peroxidation radical chain reaction and protecting RAW264.7 cells from oxidative damage (Min et al., 2018). In this study, RAW264.7 cells were pretreated with non-toxic concentrations of SBP (25, 50, and 100 μg/mL) for 4 h before the addition of H_2_O_2_ to form a protection system. As shown in Fig. 5B, compared to the control group, the SOD levels in the sample groups increased by 47.04%, 60.50% and 90.56%, respectively. At the concentration of 25 μg/mL, the SOD level of the VC group was higher than the SBP group, which was consistent with the results of the cell viability assessment. It could be concluded from Fig. 5C that with the addition of different concentrations of SBP (25, 50, and 100 μg/mL), sample groups exhibited a higher GSH-Px levels (18.59, 19.64, and 22.3 nmol/mg) compared to the control group (11.88 nmol/mg). The results also indicated that with the protection of SBP and VC, the oxidative stress caused by H_2_O_2_ was reduced significantly. This provided that SBP had superior antioxidant capacity.

The oxidation of saturated fatty acids first formed conjugated diene hydroperoxides (CD-POV), which further oxidation to form epoxides and finally decomposed to malondialdehyde and other products. With further oxidation, the decomposition of CD-POV into secondary oxidation products accelerated, and the concentration of CD-POV in the system reached a peak at first and then decreased. In contrast, the concentration of MDA kept increasing. The antioxidant effect was indicated by the concentration of CD-POV and MDA concentration or when the oxidation products’ concentration (Alyethodi et al., 2021; Zhao et al., 2021). In other words, the lower the concentration of MDA, the stronger the antioxidant capacity. According to Fig. 5D, there was a significant improvement of MDA with the treatment of 800 μmol/L H_2_O_2_ compared to the control group. Pretreated groups with SBP showed a noticeable decrease in MDA level. The results suggested that SBP had a protective effect on RAW264.7 cells and could resist the oxidative damage induced by H_2_O_2_.

#### 3.4.5 Determination of intracellular reactive oxygen species content

**Fig. 6:**
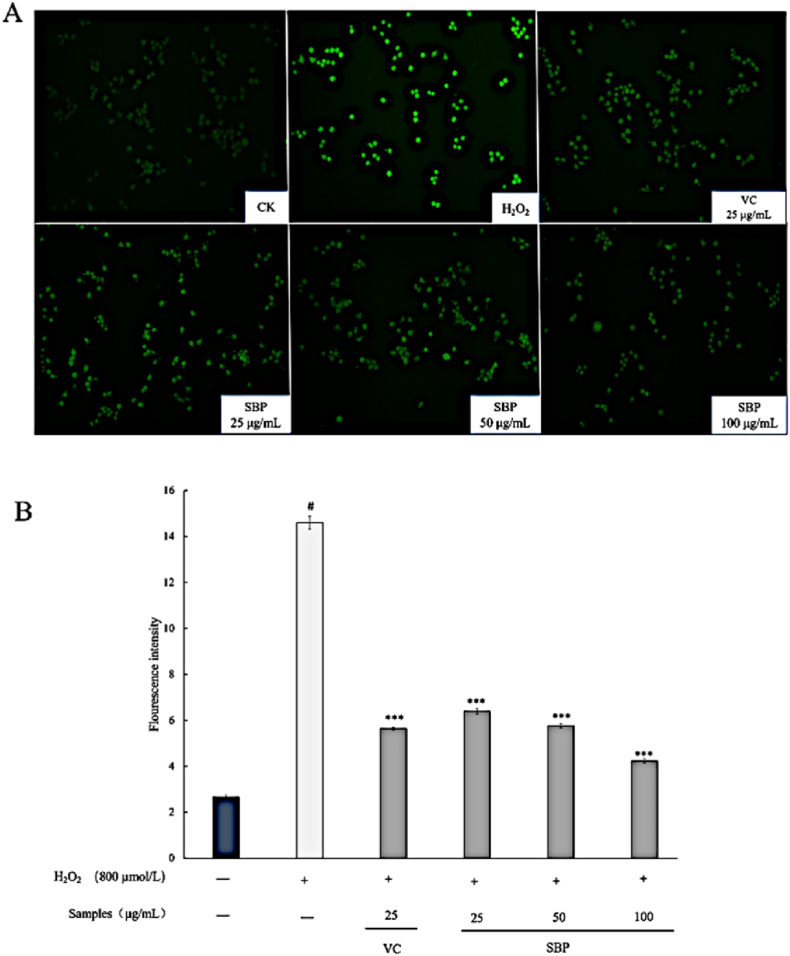
(A) Fluorescence images of cells with different treatments; (B) Intracellular ROS content of cells with different treatments.

With oxidative stress injury, dysregulation of the free radical scavenging system could lead to excessive accumulation of free radicals in the RAW264.7 cells, resulting in an excess of ROS that could attack biomolecules and caused cell damage. Therefore, a model of intracellular oxidized RAW264.7 cells was always established to assess the antioxidant capacity of compounds, particularly their intracellular ROS scavenging capacity. ROS level could be used as an indicator of cellular damage extent and the average intracellular fluorescence intensity could reflect the ROS content directly (Maya-Cano et al., 2021; Tamiji et al., 2005). In this study, 800 μmol/L H_2_O_2_ was used to incubate the RAW264.7 cells to construct the oxidative stress model, and the protected groups were treated with SBP and VC solution. The ROS content was calculated by the fluorescence light spectrophotometric value. The results in Fig. 6 showed that the damage group exhibited the highest intracellular ROS level with the brightest view. Compared with the model group, the fluorescence intensity of sample groups treated with SBP and VC were darker, indicating lower intracellular ROS content. Additionally, the SBP groups showed a significant dose-dependent decrease in ROS levels, increasing concentrations from 25 to 100 μg/mL. Thus, it could be surmised that treatments of SBP and VC conferred RAW264.7 cells protective effects against the oxidation damage induced by H_2_O_2_. Such a significant cytoprotective effect of SBP might be due to its excellent antioxidant capacity and free radical scavenging ability, which were consistent with the finding in the chemical antioxidant assays. In the study of Wang et al. (2016), similar conclusions were made about the antioxidant corn gluten peptide component. The authors’ research showed the antioxidant's ability to eliminate intracellular ROS. Their findings further confirmed the intracellular antioxidant activity of SBP.

## 4 Conclusion

In summary, SBP was extracted with hot water, and the yield and purity were 9.1% and 91.5%. The structure analysis showed SBP was an oligomeric procyanidins, mainly composed of (−)-epicatechin gallate, procyanidin B, (+)-gallocatechin-(+)-catechin and (+)-gallocatechin dimer. This research confirmed that SBP could be an excellent antioxidant in the food industry, based on the free radical (DPPH, ABTS, O_2_^•−^, ^•^OH) scavenging ability, total reduction capacity, and strong synergistic antioxidant combined with VC. The findings of the protection of SBP on RAW264.7 cells against the oxidative caused by H_2_O_2_ indicate the great intracellular antioxidant function, the increase of SOD and GSH, and the decrease of ROS and MDA levels. The results confirm that SBP had a stronger ability to resist oxidative stress and provide a basis for further research on the antioxidant properties of SBP. Future research should be devoted to exploring the specific mechanism and pathways of SBP as an antioxidant.

## Credit author statement

All authors have contributed to the manuscript.

## Data availability statement

The raw data will be made available upon request.

## Declaration of competing interest

The authors declare that they have no known competing financial interests or personal relationships that could have appeared to influence the work reported in this paper.

## References

[bib1] Alyethodi R.R., Sirohi A.S., Karthik S., Tyagi S., Perumal P., Singh U., Sharma A., Kundu A. (2021). Role of seminal MDA, ROS, and antioxidants in cryopreservation and their kinetics under the influence of ejaculatory abstinence in bovine semen. Cryobiology.

[bib2] Ashwin K., Pattanaik A.K., Howarth G.S. (2021). Polyphenolic bioactives as an emerging group of nutraceuticals for promotion of gut health: a review. Food Biosci..

[bib3] Azmi L., Shukla I., Goutam A., Rao C.V., Jawaid T., Awaad A.S., Alqasoumi S.I., AlKhamees O.A., Kamal M. (2019). Oxidative free radicals scavenging activity (*in vitro* and *in vivo* assay) of standardized fractions from the seeds of Argyreia speciosa (Ghav-patta) a traditional Indian medicine. Saudi Pharmaceut. J..

[bib4] Bai R., Cui Y., Luo L., Yuan D., Wei Z., Yu W., Sun B. (2019). A semisynthetic approach for the simultaneous reaction of grape seed polymeric procyanidins with catechin and epicatechin to obtain oligomeric procyanidins in large scale. Food Chem..

[bib5] Ciesarova Z., Murkovic M., Cejpek K., Kreps F., Tobolkova B., Koplik R., Belajova E., Kukurova K., Dasko L., Panovska Z., Revenco D., Burcova Z. (2020). Why is sea buckthorn (*Hippophae rhamnoides* L.) so exceptional? A review. Food Res. Int..

[bib7] Eroglu E., Girgin S.N. (2021). A unique phenolic extraction method from olive oil macerate of *Hypericum perforatum* using DMSO: assessment of *in vitro* anticancer activity, LC-MS/MS profile, total phenolic content and antioxidant capacity. South Afr. J. Bot..

[bib8] Friedrich W., Eberhardt A., Galensa R. (2000). Investigation of proanthocyanidins by HPLC with electrospray ionization mass spectrometry. Eur. Food Res. Technol..

[bib9] Fu C., Yang X., Lai S., Liu C., Huang S., Yang H. (2015). Structure, antioxidant and α-amylase inhibitory activities of longan pericarp proanthocyanidins. J. Funct. Foods.

[bib11] Guo R., Guo X., Li T., Fu X., Liu R.H. (2017). Comparative assessment of phytochemical profiles, antioxidant and antiproliferative activities of Sea buckthorn (*Hippophae rhamnoides* L.) berries. Food Chem..

[bib12] He Z., Zhu Y., Bao X., Zhang L., Li N., Jiang G., Peng Q. (2019). Optimization of alkali extraction and properties of polysaccharides from. Ziziphus jujuba cv. Residue. Molecules.

[bib13] Hellstrm J., Sinkkonen J., Karonen M., Mattila P. (2007). Isolation and structure elucidation of procyanidin oligomers from Saskatoon berries (*Amelanchier alnifolia*). J. Agric. Food Chem..

[bib14] Hu Y., Mo G., Wang Y., Guo J., Huang C. (2021). Fabrication and characterization of TPP-β-cyclodextrin/chitosan supramolecular nanoparticles for delivery dual bioactive compounds. J. Mol. Liq..

[bib15] Jerez M., Pinelo M., Sineiro J., Núñez M.J. (2006). Influence of extraction conditions on phenolic yields from pine bark: assessment of procyanidins polymerization degree by thiolysis. Food Chem..

[bib16] Kim S., Yang H., Lee H., Ju J. (2021). Vitro antioxidant and anti-colon cancer activities of Sesamum indicum L. Leaf Extract and Its Major Component, Pedaliin. Foods.

[bib17] Kiselova-Kaneva Y., Galunska B., Nikolova M., Dincheva I., Badjakov I. (2022). High resolution LC-MS/MS characterization of polyphenolic composition and evaluation of antioxidant activity of Sambucus ebulus fruit tea traditionally used in Bulgaria as a functional food. Food Chem..

[bib18] Li Z., Wang J., Xiong Y., Li Z., Feng S. (2016). The determination of the fatty acid content of sea buckthorn seed oil using near infrared spectroscopy and variable selection methods for multivariate calibration. Vib. Spectrosc..

[bib19] Liu G., Zhu W., Zhang J., Song D., Zhuang L., Ma Q., Yang X., Liu X., Zhang J., Zhang H., Wang J., Liang L., Xu X. (2021). Antioxidant capacity of phenolic compounds separated from tea seed oil in vitro and in vivo. Food Chem..

[bib20] Liu H., Zou T., Gao J.M., Gu L. (2013). Depolymerization of cranberry procyanidins using (+)-catechin, (-)-epicatechin, and (-)-epigallocatechin gallate as chain breakers. Food Chem..

[bib21] Luo M., Zhang R., Liu L., Chi J., Huang F., Dong L., Ma Q., Jia X., Zhang M. (2020). Preparation, stability and antioxidant capacity of nano liposomes loaded with procyandins from lychee pericarp. J. Food Eng..

[bib22] Maya-Cano D.A., Arango-Varela S., Santa-Gonzalez G.A. (2021). Phenolic compounds of blueberries (*Vaccinium spp*) as a protective strategy against skin cell damage induced by ROS: a review of antioxidant potential and antiproliferative capacity. Heliyon.

[bib23] McCord J.M., Fridovich I. (1969). Superoxide dismutase. J. Biol. Chem..

[bib59] Mekini I.G., Imat V., Boti V., Crnjac A., Skroza D. (2021). Bioactive phenolic metabolites from adriatic brown algae dictyota dichotoma and padina pavonica (dictyotaceae). Foods.

[bib24] Mihalcea L., Turturica M., Barbu V., Ionita E., Patrascu L., Cotarlet M., Dumitrascu L., Aprodu I., Rapeanu G., Stanciuc N. (2018). Transglutaminase mediated microencapsulation of sea buckthorn supercritical CO_2_ extract in whey protein isolate and valorization in highly value added food products. Food Chem..

[bib25] Min Y.N., Niu Z.Y., Sun T.T., Wang Z.P., Jiao P.X., Zi B.B., Chen P.P., Tian D.L., Liu F.Z. (2018). Vitamin E and vitamin C supplementation improves antioxidant status and immune function in oxidative-stressed breeder roosters by up-regulating expression of GSH-Px gene. Poultry Sci..

[bib26] Mizuno M., Mori K., Misawa T., Takaki T., Demizu Y., Shibanuma M., Fukuhara K. (2019). Inhibition of beta-amyloid-induced neurotoxicity by planar analogues of procyanidin B3. Bioorg. Med. Chem. Lett.

[bib27] Munoz-Labrador A., Prodanov M., Villamiel M. (2019). Effects of high intensity ultrasound on disaggregation of a macromolecular procyanidin-rich fraction from Vitis vinifera L. seed extract and evaluation of its antioxidant activity. Ultrason. Sonochem..

[bib28] Navarro-Hoyos M., Alvarado-Corella D., Moreira-Gonzalez I., Arnaez-Serrano E., Monagas-Juan M. (2018). Polyphenolic composition and antioxidant activity of aqueous and ethanolic extracts from uncaria tomentosa bark and leaves. Antioxidants.

[bib29] Odabasoglu F., Aslan A., Cakir A., Suleyman H., Karagoz Y., Bayir Y., Halici M. (2005). Antioxidant activity, reducing power and total phenolic content of some lichen species. Fitoterapia.

[bib30] Olsvik P.A., Kristensen T., Waagbo R., Rosseland B.O., Tollefsen K.E., Baeverfjord G., Berntssen M.H. (2005). mRNA expression of antioxidant enzymes (SOD, CAT and GSH-Px) and lipid peroxidative stress in liver of Atlantic salmon (Salmo salar) exposed to hyperoxic water during smoltification. Comp. Biochem. Physiol. C Toxicol. Pharmacol..

[bib31] Pagano I., Campone L., Celano R., Piccinelli A.L., Rastrelli L. (2021). Green non-conventional techniques for the extraction of polyphenols from agricultural food by-products: a review. J. Chromatogr. A.

[bib32] Quiroga P.R., Nepote V., Baumgartner M.T. (2019). Contribution of organic acids to alpha-terpinene antioxidant activity. Food Chem..

[bib33] Rue E.A., Rush M.D., van Breemen R.B. (2018). Procyanidins: a comprehensive review encompassing structure elucidation via mass spectrometry. Phytochemistry Rev..

[bib34] Ruesgas-Ramón M., Figueroa-Espinoza M.C., Durand E. (2017). Application of deep eutectic solvents (DES) for phenolic compounds extraction: overview, challenges, and opportunities. J. Agric. Food Chem..

[bib35] Sasot G., Martinez-Huelamo M., Vallverdu-Queralt A., Mercader-Marti M., Estruch R., Lamuela-Raventos R.M. (2017). Identification of phenolic metabolites in human urine after the intake of a functional food made from grape extract by a high resolution LTQ-Orbitrap-MS approach. Food Res. Int..

[bib36] Sharma K., Guleria S., Razdan V.K., Babu V. (2020). Synergistic antioxidant and antimicrobial activities of essential oils of some selected medicinal plants in combination and with synthetic compounds. Ind. Crop. Prod..

[bib60] Sierra-Cruz M., Miguens-Gomez A., Grau-Bove C., Rodriguez-Gallego E., Blay M., Pinent M., Ardevol A., Terra X., Beltran-Debon R. (2021). Grape-seed proanthocyanidin extract reverts obesity-related metabolic derangements in aged female rats. Nutrients.

[bib61] Silva A., Rosalen P.L., Camargo A., Lazarini J.G. (2021). Inajá oil processing by-product: a novel source of bioactive catechins and procyanidins from a brazilian native fruit. Food Res. Int..

[bib37] Spranger I., Sun B., Mateus A.M., Freitas V., Ricardo-da-Silva J.M. (2008). Chemical characterization and antioxidant activities of oligomeric and polymeric procyanidin fractions from grape seeds. Food Chem..

[bib38] Stalmach A., Edwards C.A., Wightman J., Crozier A. (2011). Identification of (Poly)phenolic compounds in concord grape juice and their metabolites in human plasma and urine after juice consumption. J. Agric. Food Chem..

[bib39] Su H., Li Y., Hu D., Xie L., Ke H., Zheng X., Chen W. (2018). Procyanidin B2 ameliorates free fatty acids-induced hepatic steatosis through regulating TFEB-mediated lysosomal pathway and redox state. Free Radic. Biol. Med..

[bib40] Sui Y., Zheng Y., Li X., Li S., Xie B., Sun Z. (2016). Characterization and preparation of oligomeric procyanidins from Litchi chinensis pericarp. Fitoterapia.

[bib41] Tamiji S., Beauvillain J.C., Mortier L., Jouy N., Tual M., Delaporte E., Formstecher P., Marchetti P., Polakowska R. (2005). Induction of apoptosis-like mitochondrial impairment triggers antioxidant and Bcl-2-dependent keratinocyte differentiation. J. Invest. Dermatol..

[bib42] Tang C., Xie B., Sun Z. (2017). Antibacterial activity and mechanism of B-type oligomeric procyanidins from lotus seedpod on enterotoxigenic Escherichia coli. J. Funct. Foods.

[bib43] Tkacz K., Wojdylo A., Turkiewicz I.P., Nowicka P. (2021). Anti-diabetic, anti-cholinesterase, and antioxidant potential, chemical composition and sensory evaluation of novel sea buckthorn-based smoothies. Food Chem..

[bib44] Wang J., Bie M., Zhou W., Xie B., Sun Z. (2019). Interaction between carboxymethyl pachyman and lotus seedpod oligomeric procyanidins with superior synergistic antibacterial activity. Carbohydr. Polym..

[bib45] Wang L., Ding L., Yu Z., Zhang T., Ma S., Liu J. (2016). Intracellular ROS scavenging and antioxidant enzyme regulating capacities of corn gluten meal-derived antioxidant peptides in HepG2 cells. Food Res. Int..

[bib46] Wang L., Wang C., Wang L., Zhang Q., Wang Y., Xia X. (2020). Emulsion electrospun polylactic acid/Apocynum venetum nanocellulose nanofiber membranes with controlled sea buckthorn extract release as a drug delivery system. Textil. Res. J..

[bib47] Wu Q., Feng Y., Ouyang Y., Liang Y., Zhao K., Wang Y., Luo Q., Xiao J., Feng N., Zhou M. (2021). Inhibition of advanced glycation endproducts formation by lotus seedpod oligomeric procyanidins through RAGE-MAPK signaling and NF-kappaB activation in high-AGEs-diet mice. Food Chem. Toxicol..

[bib48] Xiao P.T., Liu S.Y., Kuang Y.J., Jiang Z.M., Lin Y., Xie Z.S., Liu E.H. (2021). Network pharmacology analysis and experimental validation to explore the mechanism of sea buckthorn flavonoids on hyperlipidemia. J. Ethnopharmacol..

[bib49] Xu S.F., Zou B., Yang J., Yao P., Li C.M. (2012). Characterization of a highly polymeric proanthocyanidin fraction from persimmon pulp with strong Chinese cobra PLA2 inhibition effects. Fitoterapia.

[bib50] Yan F., Chen L., Chen W., Zhao L., Lu Q., Liu R. (2021). Protective effect of procyanidin A-type dimers against H2O2-induced oxidative stress in prostate DU145 cells through the MAPKs signaling pathway. Life Sci..

[bib51] Yang J., Chen J., Hao Y., Liu Y. (2021). Identification of the DPPH radical scavenging reaction adducts of ferulic acid and sinapic acid and their structure-antioxidant activity relationship. LWT (Lebensm.-Wiss. & Technol.).

[bib52] Yang S., Guo Z., Miao F., Xue Q., Qin S. (2010). The hydroxyl radical scavenging activity of chitosan, hyaluronan, starch and their O-carboxymethylated derivatives. Carbohydr. Polym..

[bib53] Yusoff A.H.M., Salimi M.N., Gopinath S.C.B., Abdullah M.M.A., Samsudin E.M. (2020). Catechin adsorption on magnetic hydroxyapatite nanoparticles: a synergistic interaction with calcium ions. Mater. Chem. Phys..

[bib54] Zhang H., Guo Q., Liang Z., Wang M., Wang B., Sun-Waterhouse D., Waterhouse G.I.N., Wang J., Ma C., Kang W. (2021). Anti-inflammatory and antioxidant effects of Chaetoglobosin Vb in LPS-induced RAW264.7 cells: achieved via the MAPK and NF-kappaB signaling pathways. Food Chem. Toxicol..

[bib55] Zhang J., Wang C., Wang C., Sun B., Qi C. (2018). Understanding the role of extracts from sea buckthorn seed residues in anti-melanogenesis properties on B16F10 melanoma cells. Food Funct..

[bib56] Zhao M.-T., Liu Z.-Y., Li A., Zhao G.-H., Xie H.-K., Zhou D.-Y., Wang T. (2021). Gallic acid and its alkyl esters emerge as effective antioxidants against lipid oxidation during hot air drying process of. Ostrea talienwhanensis. Lwt.

[bib57] Zhu Y., He Z., Bao X., Wang M., Yin S., Song L., Peng Q. (2021). Purification, in-depth structure analysis and antioxidant stress activity of a novel pectin-type polysaccharide from *Ziziphus Jujuba* cv. Muzaoresidue. J. Funct. Foods.

[bib58] Zou T., Li Z., Percival S.S., Bonard S., Gu L. (2012). Fabrication, characterization, and cytotoxicity evaluation of cranberry procyanidins-zein nanoparticles. Food Hydrocolloids.

